# Hydrolyzable tannins are incorporated into the endocarp during sclerification of the water caltrop *Trapa natans*

**DOI:** 10.1093/plphys/kiad408

**Published:** 2023-07-10

**Authors:** Jessica C Huss, Sebastian J Antreich, Martin Felhofer, Konrad Mayer, Michaela Eder, Ana Catarina Vieira Dias dos Santos, Georg Ramer, Bernhard Lendl, Notburga Gierlinger

**Affiliations:** Institute of Biophysics, University of Natural Resources and Life Sciences (BOKU), 1190 Vienna, Austria; Institute of Biophysics, University of Natural Resources and Life Sciences (BOKU), 1190 Vienna, Austria; Institute of Biophysics, University of Natural Resources and Life Sciences (BOKU), 1190 Vienna, Austria; Institute of Biophysics, University of Natural Resources and Life Sciences (BOKU), 1190 Vienna, Austria; Department of Biomaterials, Max Planck Institute of Colloids and Interfaces, 14476 Potsdam-Golm, Germany; Institute of Chemical Technologies and Analytics, Technische Universität Wien, 1060 Vienna, Austria; Institute of Chemical Technologies and Analytics, Technische Universität Wien, 1060 Vienna, Austria; Institute of Chemical Technologies and Analytics, Technische Universität Wien, 1060 Vienna, Austria; Institute of Biophysics, University of Natural Resources and Life Sciences (BOKU), 1190 Vienna, Austria

## Abstract

The water caltrop (*Trapa natans*) develops unique woody fruits with unusually large seeds among aquatic plants. During fruit development, the inner fruit wall (endocarp) sclerifies and forms a protective layer for the seed. Endocarp sclerification also occurs in many land plants with large seeds; however, in *T. natans*, the processes of fruit formation, endocarp hardening, and seed storage take place entirely underwater. To identify potential chemical and structural adaptations for the aquatic environment, we investigated the cell-wall composition in the endocarp at a young developmental stage, as well as at fruit maturity. Our work shows that hydrolyzable tannins—specifically gallotannins—flood the endocarp tissue during secondary wall formation and are integrated into cell walls along with lignin during maturation. Within the secondary walls of mature tissue, we identified unusually strong spectroscopic features of ester linkages, suggesting that the gallotannins and their derivatives are cross-linked to other wall components via ester bonds, leading to unique cell-wall properties. The synthesis of large amounts of water-soluble, defensive aromatic metabolites during secondary wall formation might be a fast way to defend seeds within the insufficiently lignified endocarp of *T. natans*.

## Teaser

Underwater fruit development and endocarp sclerification in *Trapa natans* involve hydrolyzable tannins in addition to lignin during cell-wall formation.

## Introduction

The water caltrop *Trapa natans* var. *natans* L. (Lythraceae; Graham and Graham 2014) is an annual aquatic plant that occurs in standing freshwater habitats in many parts of the world. The species is of conservation concern in Europe and Russia, while it is being considered a pest in North America and Australia (Hummel and Kiviat 2004). The invasiveness of *T. natans* in some regions is driven by its fast growth via clonal and sexual propagation (Groth et al. 1996; Phartyal et al. 2018). Sexual reproduction typically ensures species persistence in aquatic habitats, since vegetative reproduction is subject to high spatial and temporal fluctuations (Eckert et al. 2016). The production and storage of seeds are essential for *T. natans*, because plants usually do not survive the winter in temperate regions. Thus, the persistence of these populations depends entirely on successful recruitment from seeds. Every year, during the growing season, each floating rosette produces around 10 to 15 single-seeded fruits (Hummel and Kiviat 2004). Development of the fruits occurs underwater (Sinjushin 2018) and lasts from mid-July into October, i.e. about 3 to 4 mo in Europe and the United States (Hummel and Kiviat 2004; Borojević 2009; Seago et al. 2016). Within the clade of rosids, this is a comparatively short-time window to develop large seeds. Walnuts (*Juglans regia* L.) and pistachios (*Pistacia vera* L.), for example typically develop within 5 mo (Gijón et al. 2009; Antreich et al. 2021). The mature fruits of *T. natans* sink to the sediment, where the outermost green layer (exocarp) decomposes and the characteristic hard endocarp gets revealed. The presence of a mesocarp has not been described in the literature. In European populations, the fruit endocarp shows morphological diversity, with either 2, 3, or 4 horns (Tutin et al. 1968; Frey et al. 2017). The samples from our study region in lower Austria typically have 4 horns with sharp barbed spines attached. The persistent endocarp may resist decomposition for up to 10 yr and serves as an anchor for the seed during dormancy, germination, and growth (Hummel and Kiviat 2004; Seago et al. 2016). In order to break the physiological dormancy of the seeds, mature fruits require cold stratification (4°C) for a minimum of 9 wks (Phartyal et al. 2018). Seeds are still viable after 12 yr in the sediment, although most typically germinate within the first 2 yr (Swearingen et al. 2010). Based on the short life cycle of this annual plant, it is evident that the endocarp plays a crucial role for persistence in the aquatic habitats of *T. natans*. Despite the biological importance, it remains largely unknown how the tissue anatomy and composition compare with those of other lignocellulosic shells, such as walnuts (*J. regia*) or pistachios (*P. vera*).

In this study, we identify essential anatomical and biochemical features within the developing and mature endocarp tissue of *T. natans*. Our work discusses the developmental changes in cell-wall structure and composition and how these parameters may contribute to endocarp durability underwater.

## Results

### Fruit development and morphology

**Figure 1.**
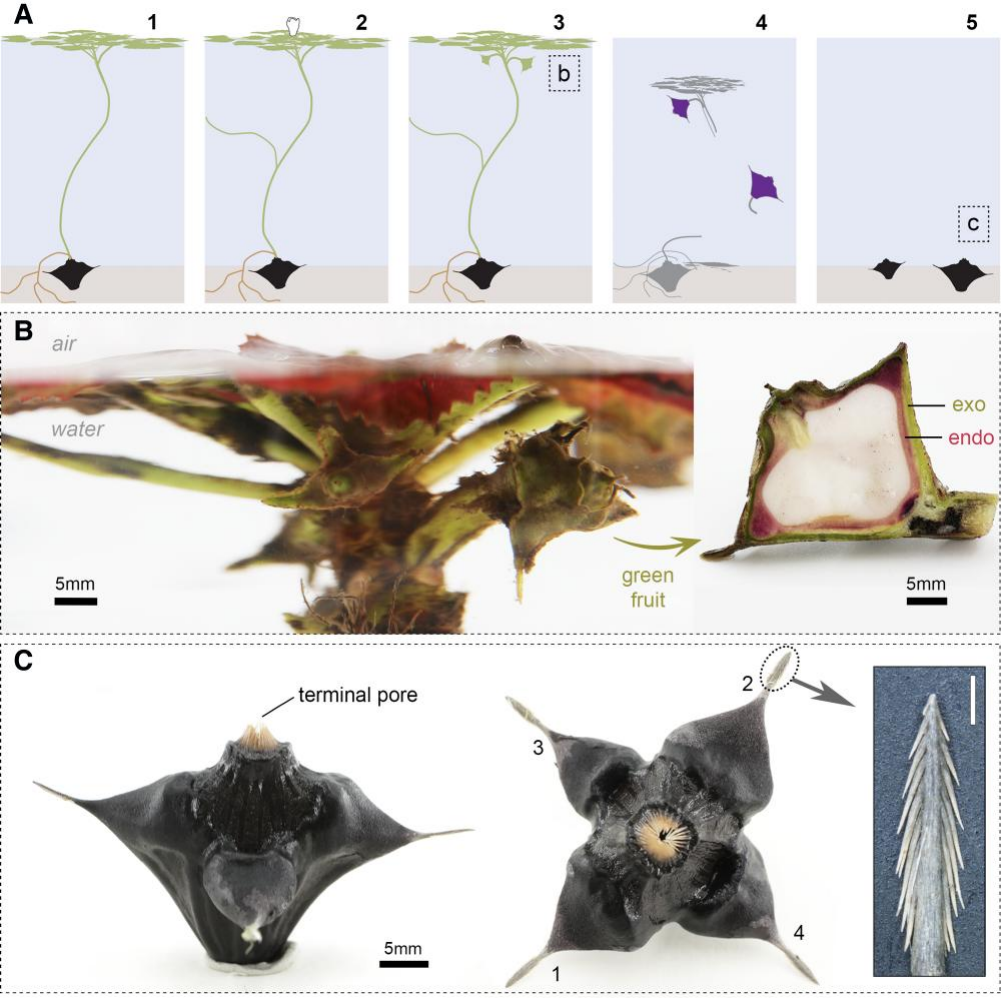
Morphology of *T. natans* in different developmental stages. **A)** Schematic illustration of the annual growth cycle, including (1) germination and formation of a rosette on the water surface in spring; (2) flowering and vegetative propagation by branching (ramets); (3) submergence of the pollinated flowers and nut development underneath the rosette (developmental stage shown in Panel b); (4) nut maturation and plant senescence in autumn; (5) seed dormancy and nut storage on the sediment during winter (fruit shown in Panel c). **B)** In mid-September, the leaves of the floating rosette started to change color from dark green to an intense red, and fruits of variable sizes and developmental stages can be seen below the water surface. The longitudinal cut of a developing fruit (referred to as “green fruit”) shows the fully developed seed surrounded by the pink endocarp (endo) and green, fleshy exocarp (exo) (see Supplemental Movie S1). **C)** During seed storage in winter, the green exocarp decomposes and reveals the now dark endocarp (nuts were sampled in March). The side and top views of a mature (wet) fruit show the terminal pore and 4 barbed spines. The 2 upper spine extensions (1,2) are larger and accommodate the seed, while the lower 2 (3,4) are smaller and can be absent in some populations (Frey et al. 2017). Inset: Magnification of the horn tip reveals the fine and sharp barbs; scale bar: 1 mm.

The fruits of *T. natans* develop underwater, and each of them typically contains a single seed (Fig. 1, A to C). During development, the submerged fruits are covered by a green exocarp and connected to the central floating rosette by the pedicel (Fig. 1, A and B). From our observations in mid-September, individual plants carry fruits in different developmental stages and sizes simultaneously, but the majority had already fully developed seeds. This aligns well with the observation of consecutive flowering by Sinjushin (2018). The mature fruits collected in spring display an opening that is covered by a hairy dome, resembling a filter or a valve (Fig. 1C). Sinjushin (2018) termed the opening “coronary disk,” and Hummel and Kiviat (2004) described it as “terminal pore.” During germination, the radicle emerges through this opening (Tutin et al. 1968). Due to the directed arrangement of the fibers, the structure facilitates outward movements, while simultaneously limiting inward movements (i.e. into the capsule), for example penetration by small soil particles. This open capsule structure allows for constant seed hydration in water and appears to be a clear adaptation to the aquatic habitat. Many studies reported that the seeds (within the endocarp) are sensitive to dehydration (e.g. Phartyal et al. 2018), which can easily be explained by the presence of the terminal pore, leading to fast drying of the seed when diaspores are removed from water.

### Chemical impregnation of cell walls during development

**Figure 2.**
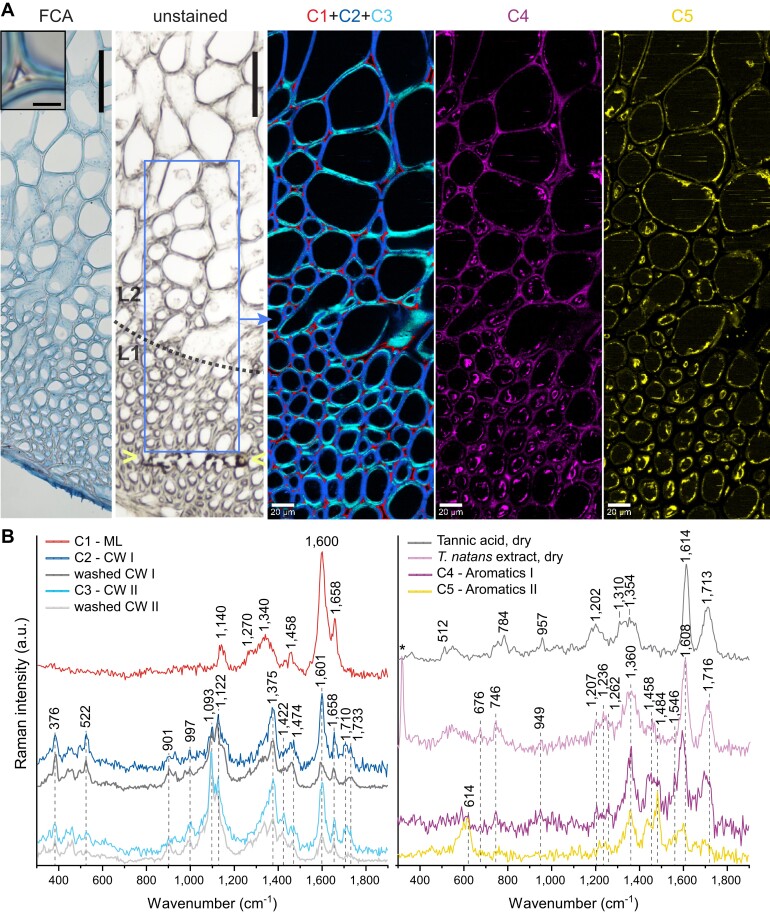
Imaging of young endocarp tissue from green fruits. **A)** FCA-stained tissue shows that the middle lamella is already lignified (inset: magnified cell corner; scale bar: 4 *μ*m). Unstained tissue indicates the measurement area for Raman imaging (blue box) and the burned area (markers) near the edge of the tissue, which has been caused by the laser. Scale bars: 100 *µ*m. Raman images were obtained via True Component Analysis, showing the intensity distribution and the respective spectra of the identified components: middle lamella (C1-ML) and cell wall compounds (C2-CW I and C3-CW II), as well as aromatic components in the cell wall (C4-Aromatics I) and in the lumen (C5-Aromatics II). C1 + C2 + C3 is a composite image. **B)** Raman spectra of individual components and reference compounds. Spectral features of C4 overlap largely with those of the pink extract and show similarities to the reference compound tannic acid. The measurement of the washed section is shown in Supplemental Fig. S3. The peak at 318 cm^−1^ (*) in the Raman spectrum of the pink extract arises from the CaF_2_ slide (Supplemental Fig. S4). All Raman measurements were performed with a *λ* = 532 nm laser excitation. FCA, Fuchsin-Chrysoidin-Astrablue; C, component; L, layer; ML, middle lamella; CW, cell wall.

The endocarp of green fruits is characterized by an intense pink coloration, especially in the horn region and at the base near the pedicel (Fig. 1B, Supplemental Movie S1). However, tissue sections lose color immediately when wetted, suggesting that these compounds are water-soluble. Histochemical staining and Raman spectroscopy measurements across the outermost (L1) and central layer (L2) in the developing horn tissue allowed us to understand the chemical changes during sclerification in more detail (Fig. 2A). While the middle lamella is already fully lignified in mid-September, the secondary cell-wall layers are still being formed and undergo lignification. In the identified Raman spectra (Fig. 2), this is visible for component C1 (corresponding to the middle lamella), which shows typical bands of lignin (guaiacyl dehydrogenation polymer) at 1,656; 1,598; 1,458; 1,335; and near 1,140 cm^−1^ (Bock et al. 2020). The secondary cell-wall layers clearly show a variable orientation of cellulose microfibrils (Fig. 2B, C2 to C3), which is reflected in different intensities of the orientation-sensitive band at 1,093 cm^−1^ (e.g. Gierlinger et al. 2010; Felhofer et al. 2021). Based on the locally enhanced band intensity in individual lamellae, we conclude that the cellulose microfibril angle (MFA) varies between lamellae of the S2 cell-wall layers (Supplemental Fig. S1). The preferential orientation of cellulose microfibrils in different secondary wall layers of plants is consistent with previous observations in wood (Barnett and Bonham 2004) and aids in load transfer from micro- to mesoscale and vice versa. For the endocarp of *T. natans*, a variable MFA between lamellae of cell walls may contribute to the mechanical reinforcement of the tissue in a water-saturated state, which is particularly important for the thin-walled regions, e.g. in the central layer of the horn (L2, Fig. 2A). In addition to lignin and cellulose, we identified 2 other components (C4 and C5). The intensity of C4 is the strongest near the S1 cell-wall layer and at the interface between cell wall and lumen (Fig. 2A). The Raman bands of C4 correspond well with those of the dried, pink extract from young fruits and tannic acid, but show additional bands in the CH-stretching region with a maximum at 2,936 cm^−1^, which arise from cell-wall components. Due to spectral similarities in the fingerprint region (Fig. 2B), we conclude that C4 corresponds to the aqueous extract from the tissue, which shares distinct marker bands with gallotannins, i.e. tannic acid. These results are also confirmed by a detailed mixture analysis of cell-wall spectrum C2 (Supplemental Fig. S2). Component 5 (C5) is mainly present in the lumen and differs in its Raman bands and background signal (stronger fluorescence) compared to C4. Based on the spectral bands and its spatial intensity distribution, C5 has to be assigned to other compounds, presumably compounds of the protoplast, such as proteins, lipids or secondary metabolites.

The aqueous extract from young endocarp tissue was characterized by pink coloration and formed a gel-like film when air-dried. Due to the water solubility and distinct bands in the Raman spectrum, we conclude that hydrolyzable tannins, in particular gallotannins, are flooding the tissue during secondary wall formation. The gallotannins flow primarily through the vasculature (Supplemental Movie S1) and through pit channels in the central tissue layer L2 during development (Fig. 2A). As shown by Raman imaging, the compounds directly interact with cell walls during their formation. However, at this stage, the gallotannins largely occur unbounded in cell walls and can be dissolved by polar solvents, because the characteristic bands (aromatic ring stretching band at 1,603 cm^−1^ and C=O stretching vibrations of esters from 1,700 to 1,715 cm^−1^) are strongly reduced in cell-wall spectra from sections that have been washed with a mixture of ethanol and water prior to measurements (Fig. 2B, Supplemental Fig. S3). Based on the changes in band intensity after washing and the presence of gallotannins in secondary walls in nonwashed sections, we conclude that the developing walls directly interact with gallotannins due to their hydrophilic properties.

For further analysis, we used the prominent marker band of the pink extract at 745 cm^−1^ in its Raman spectrum, which is particularly strong when measured with the 785 nm laser (in contrast to the laser excitation with a wavelength of 532 nm). This spectral band is suitable for identifying the extract within the tissue, because this region is mostly free from overlapping bands of typical cell-wall compounds, e.g. cellulose does not show any bands here at all.

### Composition of cell walls in the mature state

**Figure 3.**
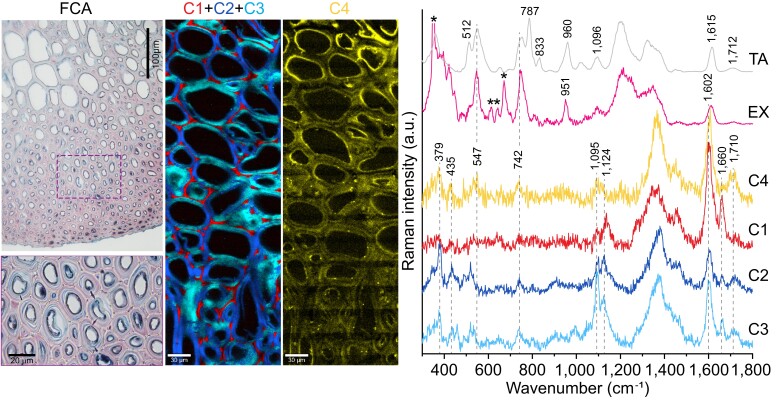
Imaging of mature endocarp tissue in the horn region across L1 and L2. FCA staining indicates lignified cell walls in red (box indicates a higher magnification region shown at the bottom). Raman images of washed sections were obtained from True Component Analysis and show the middle lamella (C1), cell walls with different orientations of cellulose (C2 and C3), and aromatic compounds located in cell walls and a high intensity toward the lumen (C4). C1 + C2 + C3 is a composite image. The corresponding spectra are shown along with TA and the dried aqueous extract from young endocarp tissue (EX, asterisks indicate bands from the CaF_2_ sample holder, the band at 350 cm^−1^ has been cut off for better scaling). The strong signal around 1,380 cm^−1^ in C1 to C4 originates from D_2_O. All Raman measurements were performed with a λ = 785 nm laser excitation, resulting in a stronger intensity of bands in the lower wavenumber region (ʋ < 1,000 cm^−1^, compared with spectra obtained with the λ = 532 nm laser) and an overall lower fluorescence background. FCA, Fuchsin-Chrysoidin-Astrablue; C, component; TA, tannic acid; EX, extract.

The mature cell walls show thicker secondary walls with stronger lignification (Fig. 3) compared with the younger state (Fig. 2). However, the density gradient between L1 and L2 remains in the horn tissue. Raman imaging of mature cell walls shows again that the middle lamella is lignified and cellulose orientation changes between different lamellae of the S2 wall (Fig. 3). In contrast to the young developmental state, the spectra of mature secondary cell walls indicate that the gallotannins previously identified are still present in cell walls, even after washing sections with a solution of ethanol and water. In cell-wall spectra, the presence of gallotannins can be identified due to a consistent broad band near 742 cm^−1^, which is slightly shifted in spectra of the pink extract—showing a strong and broad band at 748 cm^−1^. In addition, the spectra of mature cell walls show clear ester bands in the spectral range from 1,698 to 1,740 cm^−1^ (both in washed and native sections), suggesting that the gallotannins are now bound to cell-wall components via ester linkages and thus, cannot be dissolved anymore. This is supported by the very low band ratio of 1,710 vs. 742 cm^−1^ for the pink extract when compared with the ratio in mature cell walls (C2 to C4 in Fig. 3), indicating that the dissolved gallotannins have a smaller number of ester linkages compared with wall-bound gallotannins. A similarly low ratio is also observed in the water-soluble reference compound tannic acid; showing that the spectral changes are consistent in both compounds when employing a 785 nm laser excitation.

**Figure 4.**
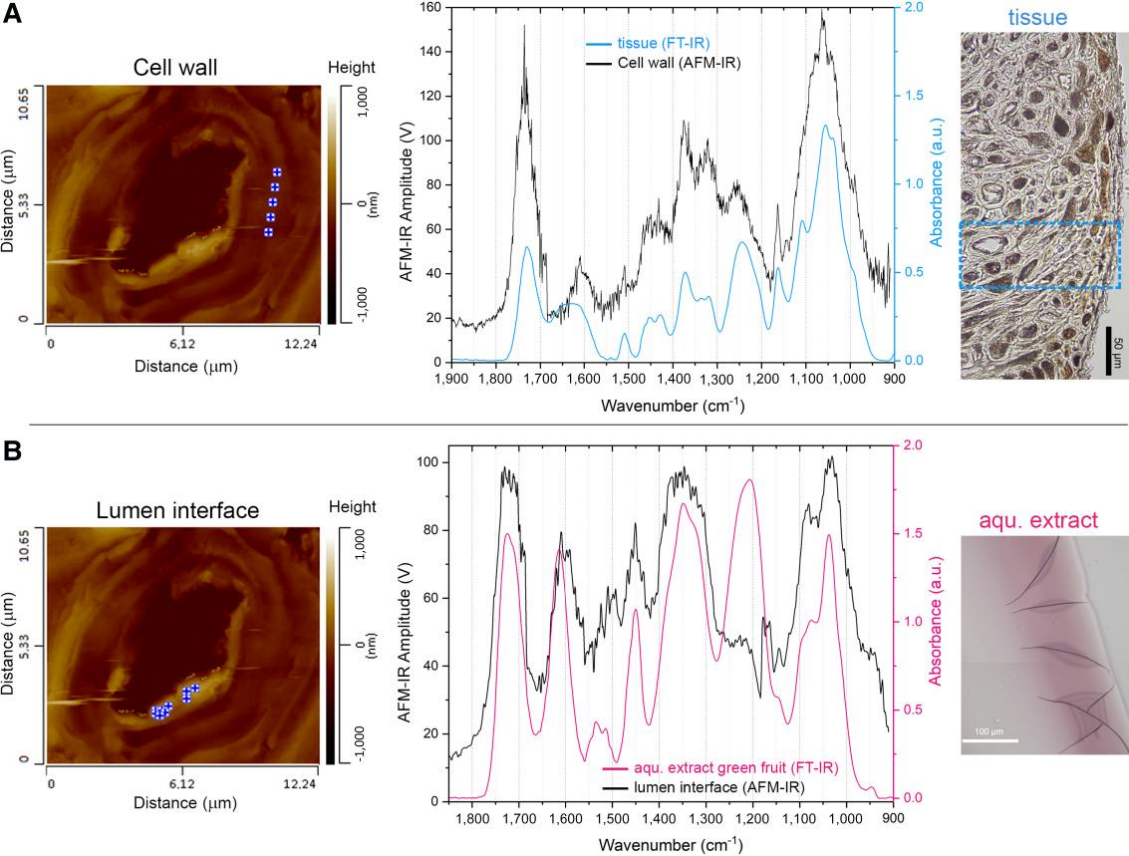
Characterization of mature cell walls via AFM-IR and FT-IR microscopy. **A)** Contact mode AFM image of 1 cell with measurement points on the secondary cell wall; corresponding AFM-IR and FT-IR spectra; as well as a light microscopy image of the outer endocarp layer (unstained) near the horn base, showing exemplary FT-IR measurement area. **B)** Contact mode AFM image of the same cell with measurement points on the interface between lumen and secondary cell wall; corresponding AFM-IR and FT-IR spectra are shown, as well as 2 overlapping light microscopy images of the edge of the film that formed from the aqueous (aqu.) extract. The band of the extract at ∼1,200 cm^−1^ is absent in the AFM-IR spectrum due to a laser transition in this region. AFM-IR, atomic force microscope-infrared spectroscopy; FT-IR, Fourier-transform infrared spectroscopy.

Mature cell walls have also been measured with Atomic Force Microscopy—Infrared (AFM-IR) and Fourier-transform Infrared (FT-IR). Their spectra (Fig. 4) show similar signatures as previously identified with Raman spectroscopy, namely strong cellulose bands at 1,162; 1,109; and 1,056 cm^−1^, as well as lignin bands at 1,507 and 1,590 to 1,600 cm^−1^ that are both assigned to aromatic ring stretching (Bock et al. 2020). In comparison with other lignocellulosic nutshell tissues, i.e. walnut and pistachio (Supplemental Fig. S5), the relative intensity and spectral contributions of lignin bands for *T. natans* are similar to those of pistachio shells (*P. vera*; Supplemental Fig. S5). Therefore, we suppose that the lignin content in the endocarp tissue of *T. natans* (in L1 to L2) may be similar to that of pistachio shells, which contain about 17% of lignin (Landucci et al. 2020). However, the cell walls of *T. natans* show more complex absorption bands with contributions of both lignin and gallotannins, as previously identified. On the cell-wall level, the composition differs locally, which is well illustrated by the AFM-IR spectra of the secondary cell-wall layer (Fig. 4A) when compared with the interface between cell wall and lumen in L1 (Fig. 4B). The spectral features of the interfacial layer correspond well with those of the FT-IR spectrum of the pink extract from green fruits (Fig. 4B), confirming that the gallotannins remain in the tissue post maturation.

### Transport pathways of gallotannins in the endocarp

**Figure 5.**
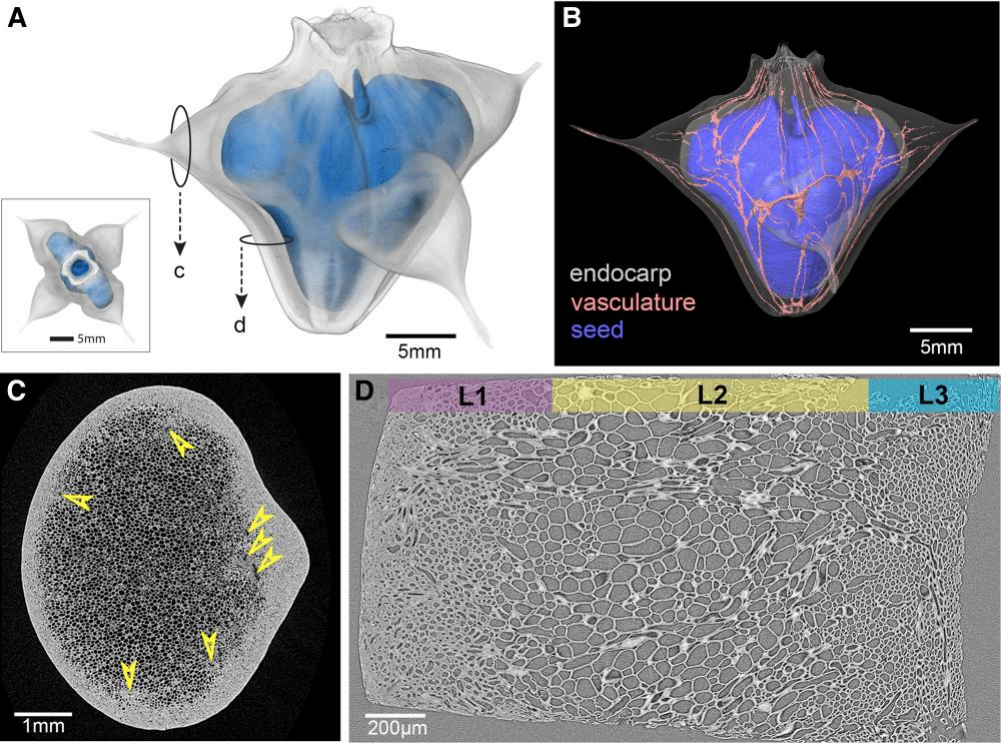
Overview of the endocarp anatomy in the mature state by means of X-ray computed microtomography. **A)** 3D image of the seed within the endocarp, *inset* shows top view highlighting the open structure of the terminal pore; **B)** 3D image of the branching vasculature within the endocarp, surrounding the seed. **C)** 2D image showing the density gradient in the horn tissue and arrowheads highlighting the vascular bundles; and **D)** the 3 distinct layers (L1, L2, and L3) showing different tissue densities (cell wall thickness and cell size) in the lateral wall of the endocarp.

The developing endocarp tissue is flooded by gallotannins that are distributed via a conspicuous network of vascular bundles (Fig. 5). This branching network pervades the endocarp from its base to the terminal pore (Fig. 5, A and B) and includes side branches of vasculature into the horns (Fig. 5C). The bright fibers that form the filter-like valve structure seem to be connected to or emerge from the vascular bundles in the endocarp. In general, the endocarp can be subdivided into 3 layers based on the cell type and cell-wall thickness (Fig. 5D): a dense layer of thick-walled fibrous cells with agglomerates in the lumina forms the outermost layer (L1); followed by a central layer consisting of large, thin-walled and nearly isodiametric cells with large intercellular spaces (L2), and an inner layer surrounding the seed (L3), which consists of thin-walled fibers with varying orientations. The vasculature is mainly located within tissue layer L2, which facilitates symplastic transport due to the large cells with thin walls and many cell–cell connections via pit channels (Fig. 2A). Additionally, the gallotannins could also be identified within the cell walls of young and mature tissue (Figs. 2 to 4). Therefore, apoplastic transport also occurs, but is likely to be more pronounced in the young stage, while secondary cell walls are not yet fully lignified.

### Chemical composition of the gallotannins

**Figure 6.**
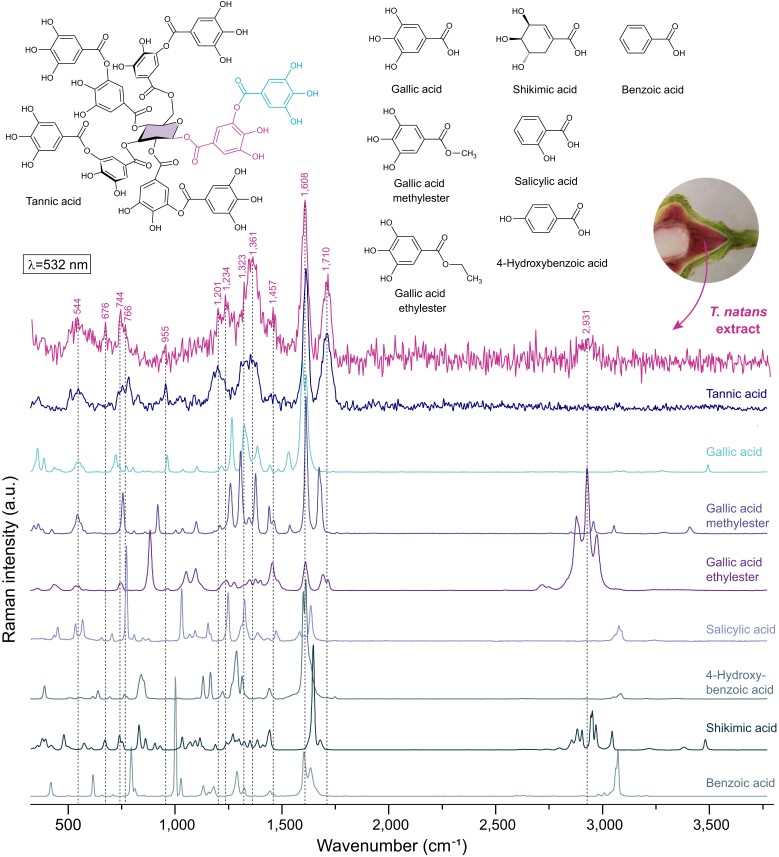
Overview of the chemical structure and Raman spectra of reference compounds with common structural features of gallotannins, their biosynthetic precursors and derived compounds. The galloyl units in the structure of tannic acid are highlighted. The spectrum of the pink extract from young endocarp tissue shares all major bands with tannic acid in the fingerprint region and can therefore be classified as a gallotannin. Minor mismatches in relative band intensity and the presence of few additional bands indicate that the extract could be a mixture of gallotannins with different numbers of galloyl units or that some residual moieties differ structurally.

The chemical structure of gallotannins is characterized by a central glucose unit esterified with at least 6 galloyl groups, including 1 or more digalloyl groups (Salminen and Karonen 2011). When inspected with Raman and FT-IR spectroscopy, the bands of the pink extract fit well with those of tannic acid (Figs. 2, 3, and 6), which is typically described as having 5 digalloyl groups. However, minor mismatches of peaks indicate that the extract of *T. natans* may be composed of a different mixture of gallotannins or derivatives thereof. In addition to a variable number of gallic acid moieties, Huang et al. (2016) reported caffeoyl, nicotinoyl, and hexahydroxydiphenoyl units in extracted gallotannins of mature endocarps analyzed by NMR. Since gallotannins are synthesized in the shikimate pathway, we compared the spectral features of gallotannins from the pink extract along with typical precursors and closely related compounds that could occur as a mixture or as esterified moieties (Fig. 6). The extract, as well as tannic acid share many bands with gallic acid, but clearly show strong differences in the spectral region of esters (1,700 to 1,730 cm^−1^). Depending on the length of the hydrocarbon chain attached to the gallic acid unit, strong bands arise in the CH-stretching region around 2,931 cm^−1^, clearly visible for gallic acid ethylester. The positioning and number of hydroxyl groups attached to the phenol ring also have a very strong effect on the spectral features, as demonstrated by salicylic acid, 4-hydroxybenzoic acid, benzoic acid and shikimic acid: the position of the aromatic ring stretching vibration shifts to higher wavenumbers with a larger number of ring substitutions (band at 1,603 cm^−1^ in benzoic acid and at 1,645 cm^−1^ in shikimic acid). Furthermore, the position of a single hydroxyl group on the phenol ring has a strong effect on all bands, but cannot explain the mismatch of some bands between tannic acid and the extract. Ring substitution with methyl instead of hydroxyl groups results in a better match of major bands, e.g. those of 2,3-dimethylbenzoic acid (Supplemental Fig. S6) with characteristic bands at 745; 1,268; 1,447; and 1,611 cm^−1^, as well as at 2,924 cm^−1^.

## Discussion

The endocarp of *T. natans* undergoes substantial biochemical changes during development and maturation, which are characterized by 2 important co-occurring processes: the cell walls become impregnated with (i) gallotannins and (ii) lignin. Gallotannins accumulate predominantly in the secondary cell walls and at the interface toward the lumen, whereas lignin accumulates in the middle lamella and in secondary cell walls. Both types of aromatic compounds are produced in the shikimate pathway (Kause et al. 1999; Niklas et al. 2017) and their formation overlaps temporally and spatially during tissue development in *T. natans*. Based on the strong spectral features of ester bonds in the mature cell walls (Fig. 3), we conclude that the gallotannins are integrated into cell walls via ester bonds. The detailed structure of the gallotannins varies depending on the developmental stage (dissolvable vs. wall-bound) and might be affected by potential chemical modifications during extraction. In addition, as discussed by Salminen and Karonen (2011), when using gallotannins as reference compounds, e.g. tannic acid, one should consider their natural heterogeneity, as well as impurities from closely related compounds.

In other plant species, the integration of gallotannins into cell walls has previously been investigated by Grundhöfer and Gross (2001), showing that gallotannins can be bound into the cell walls of leaves in oak (*Quercus robur*), sumac (*Rhus typhina*), and fringe cups (*Tellima grandiflora*). It was suggested that the incorporation of gallotannins into cell walls may lead to an increased toughness and durability (Kause et al. 1999). However, this has not been shown experimentally. Grabber et al. (2012) showed that some monomers with structural similarity to monolignols, such as gallic acid ethylester and pentagalloyl glucose, can act as substitutes during lignification and form wall-bound lignin. In in vitro experiments of their study, both compounds produced lower lignin concentrations compared with treatments with the classical monolignols (coniferyl and sinapyl alcohol), possibly due to their capacity to modulate cross-linking with polysaccharides. The interactions between carbohydrates and aromatic compounds in cell walls have recently been studied by solid-state NMR, showing that polysaccharides, such as cellulose and xylan, can associate directly with aromatic polymers, such as lignin (Kirui et al. 2022). Compared with lignin, gallotannins presumably associate more frequently with cell wall polysaccharides due to their more hydrophilic properties. We observed this in the cell walls of *T. natans*, where the gallotannins are only found in developing and mature secondary cell walls, but were undetectable in the lignin-rich middle lamella. It is only in the young stage that the gallotannins can be dissolved from cell walls by using a mixture of water and ethanol. This indicates that the gallotannins become immobilized by interactions with other wall components during maturation.

In fruits of the genus *Trapa*, the presence of hydrolyzable tannins has repeatedly been reported: in mature endocarps of *Trapa* spp., a high content has previously been identified in *T. natans* (Huang et al. 2016; Lü et al. 2022); in *Trapa bispinosa* (Iwaoka et al. 2021); in *Trapa quadrispinosa* (Liang et al. 2020); and in *Trapa japonica* (Yasuda et al. 2014). The production and storage of hydrolyzable tannins have been associated with plant defence against insect and microbial attacks. Tuominen (2013) and Tuominen et al. (2013) reported that galloyl glucose is effective against herbivory in the wood cranesbill (*Geranium sylvaticum*). The hydrolysis product gallic acid has antifungal properties (Zhu et al. 2019) and is known to inhibit insect development in all stages (Sharma and Sohal 2013; Punia et al. 2021). Antimicrobial and antioxidant properties have been reported for gallotannins with different numbers of galloyl groups (Buzzini et al. 2008; Engels et al. 2009; Daglia 2012) and were found in the endocarp of mango (*Mangifera indica*) and in the reaction zone of pruning wounds in the shining gum (*Eucalyptus nitens*; Barry et al. 2001; Luo et al. 2014). Furthermore, gallotannins have also been associated with the red coloration in red sword bean (*Canavalia gladiata*) seed coats (Gan et al. 2018).

Compared with nutshell tissues of terrestrial plants (walnut and pistachio), the mature endocarp of *T. natans* shows a more complex composition with clear indications of incorporated gallotannins. The reasons for these differences remain to be studied, but could be related to the aquatic habitat and the short life cycle of the plant, which may benefit from an increased production of aromatic metabolites from the shikimate pathway. The dynamics of the shikimate pathway in plants are affected by environmental conditions, i.e. changes in light availability, microbial attacks or herbivory, and can lead to a higher production of aromatic metabolites under stress (Weaver and Herrmann 1997). The biosynthesis of gallotannins is driven by enzymatic reactions that occur early on in the shikimate pathway. Lignin biosynthesis, in contrast, requires many more enzymatic reaction steps and production pathways (Maeda and Dudareva 2012; Mottiar et al. 2016). Therefore, in the case of *T. natans*, the production of gallotannins might be a fast way to synthesize a large amount of defensive compounds while lignin concentrations are not yet fully sufficient to protect the seeds within the endocarp.

## Materials and methods

### Sampling

Fruits of water caltrops (*T. natans*) were collected on March 30th 2018 (mature), on September 14th 2021 and on September 23rd 2022 (green) from a pond of a private grower in Klosterneuburg (lower Austria) and from a wild population near Drösing in lower Austria (with an official collection permit). All fruits were transported in the hydrated state, representative samples photographed and stored in the freezer until further use.

### Photographs

Water caltrops were photographed with a macro camera (EOS M10, Canon; objective EFS 35 mm). For imaging of the spines, a stereomicroscope (SMZ800, Nikon) was used.

### Cryostacking

Frozen nuts from September (green fruits) were sliced with a thickness of 100 *μ*m in a cryostat at −15°C. After every cut, an image was taken from the remaining surface using a macro camera (EOS M10, Canon; objective ERF 35 mm) mounted to the cryostat. The photos were then processed and aligned in ImageJ (version 1.52p, NIH) using linear stack alignment with SIFT.

### Histochemical staining

For staining, 10 to 12 *μ*m thick sections were obtained from a cryomicrotome (Cryostat CM 3050 S, Leica) at −12°C and stained with fuchsin-chrysoidin-astrablue (0.1 g fuchsin, 0.143 g chrysoidin and 1.25 g astrablue dissolved in 1,000 mL aqua dist. and 20 mL acetic acid) for 30 min. Sections were then sequentially rinsed with aqua dist., 30% v/v EtOH, 70% v/v EtOH and 100% v/v isopropyl alcohol, followed by embedding in Euparal (Roth). Images were then taken on a light microscope (Labphot-2, Nikon).

### Aqueous extraction

The outer green layer was removed from 2 horns of green fruits using a razor blade and around 50 slices with a thickness of 50 *μ*m were cut from the young endocarp tissue with a cryostat at −12°C. Slices were collected in a small glass tube and a few drops of distilled water were added. After 45 min of shaking, the solution was filtered through a syringe with a hydrophobic 0.2 *μ*m PTFE filter and a droplet was placed on a CaF_2_ slide (10 mm × 0.35 mm polished window, Crystran Ltd.) and left to evaporate, forming a thin, pink film (Fig. 4B).

### X-ray computed microtomography

Entire nuts were scanned with a Hamamatsu micro-focus tube in an EasyTom 150/160 system (RX solutions) with a flat panel detector. Scan parameters were set to a current of 200 *μ*A, a voltage of 40 kV and an exposure time of 0.5 s with a frame averaging of 3. The collected projections had a voxel size of 22.59 *µ*m. A small cube of tissue was also scanned with the same system using a Hamamatsu nano focus tube (tungsten filament); set to a current of 200 *μ*A, a voltage of 62 kV and a voxel size of 1.19 *µ*m. For image reconstruction, we used the software XAct 2 (RX solutions). 3D visualization and segmentation were performed with the 3D software Amira (version 6.01, FEI) and ImageJ (version 1.52, NIH).

### Raman microspectroscopy

Cross sections of the horn were cut with a thickness of 12 *μ*m on a cryostat (CM 3050 S, Leica) at −12°C, transferred onto a glass slide and immersed in D_2_O or H_2_O, then sealed with nail polish. Some sections of young and mature tissue were washed on top of an objective slide with a solution of ethanol and water (1:1), then immersed in D_2_O or H_2_O and sealed with nail polish. Measurements were performed on a confocal Raman microscope (alpha300RA, WITec) with a 100× oil objective (NA = 1.4, Carl Zeiss) using 2 different lasers (532 and 785 nm). For measurements with the 532 nm laser, the samples were excited with a linear polarized Sapphire SF laser (Coherent, USA). Scattering was detected with an optic multifiber (50 *μ*m) directed to a spectrometer (UHTS-300 VIS, WITec, Germany) set to a 600 g mm^−1^ grating and connected to a CCD camera (Andor DV401 BV, Belfast, Northern Ireland). Measurements with the 785 nm laser were performed on the same instrument (with the same grating and CCD camera), using a diode laser (XSL3100-1174, WITec, Germany), an optic multifiber (100 nm) and a spectrometer (UHTS-300 NIR, WITec, Germany). Young tissue and the references were measured with the 532 nm laser at full laser power (40 mW) with an integration time of 0.05 s, while mature fruit tissue and relevant references were measured with the 785 nm laser, also at full power (201 mW) with an integration time of 0.05 s. The laser power was measured with a power meter (PM100D, Thorlabs, Germany). For data analysis, we used WITec Project 5 PLUS with the integrated True Component Analysis Tool using baseline-corrected spectra in the spectral range from 220 to 2,000 cm^−1^ (for measurements with 532 nm) and 215 to 1,812 cm^−1^ (for measurements with 785 nm) with negative weighing factors allowed. Individual components were exported to WITec TrueMatch (version 6) and compared with our in-house reference substances and the ST Japan 5.2 Database for matching compounds.

### Fourier-transform infrared spectroscopy

#### ATR mode

An FT-IR spectrometer (Vertex 70, Bruker Austria) was used to obtain spectra of pure reference substances from an ATR unit. For the references, average spectra of 5 measurements (each with 32 scans and a spectral resolution of 4 cm^−1^) were cut and baseline corrected (polynomial with a concave rubber band, 1 iteration) with OPUS 7.5 software (Bruker). For plotting, all spectra were min-max normalized.

#### Microscopy (transmission mode)

From the horn, longitudinal sections with a thickness of 6 *μ*m were cut with a cryostat, placed on top of a CaF_2_ window and left there until dry. The same procedure was applied for walnut (*J. regia*) and pistachio (*P. vera*) shells. A droplet of the aqueous extract was also placed on top of a CaF_2_ window and left to dry before measurements were performed. Since the supporting window absorbs at wavenumbers ʋ < 900 cm^−1^, this spectral range was omitted. Measurements were performed in transmission mode on a microscope (Hyperion 2000, with a liquid nitrogen cooled MCT-D316-025 detector, Bruker) coupled to the spectrometer (VERTEX 79, Bruker Austria) with a spectral resolution of 2 cm^−1^ and 32 scans per spectrum. Absorbance spectra were acquired and then baseline corrected with OPUS (version 7.5, Bruker) using the polynomial fit with a concave rubber band and 1 iteration.

#### AFM-IR

For the measurements, samples sections were cut on an ultramicrotome with a glass knife and a thickness of 400 nm, put on top of a ZnS window (13 mm diameter, 1 mm thick, Crystran Ltd.) with a drop of distilled water and left to evaporate. AFM-IR measurements were carried out using a Bruker nanoIR 3 s coupled to a MIRcat-QT external cavity quantum cascade laser array (EC-QCL) from Daylight Solutions. Spectra covering the range from 910 to 1,975 cm^−1^ (QCL transitions at 1,182; 1,520; and 1,685 cm^−1^) were obtained using AFM-IR in resonance-enhanced contact mode. The repetition rate of the laser was tuned to match the second contact resonance frequency of the cantilever (roughly 200 to 230 kHz) and the sample. The cantilevers used were gold-coated with nominal first free resonance frequencies of 13 ± 4 kHz and a nominal spring constant between 0.07 and 0.4 N m^−1^ (ContGB-G from BudgetSensors). The laser source operated at 3% duty cycle and the laser power was adjusted to 14.75% of the original power (before beam splitter, nominal splitting ratio 1:1). The instrument and all beam paths were purged with dry air generated by an adsorptive dry air generator. For each location, 5 spectra were recorded at 1 cm^−1^ spectral resolution. The obtained AFM-IR spectra were averaged by location and smoothed (2 pt intensity) in the software Analysis Studio (version 3.15, Anasys Instruments).

## Supplementary Material

kiad408_Supplementary_Data

## Data Availability

The article's supporting information can be accessed via the Journal's website (Supplemental Information); large data sets (CT scans) are accessible in Zenodo (doi:10.5281/zenodo.7097856).

## Supplemental data

The following materials are available in the online version of this article.

**Supplemental Movie S1.** Cryostack of a green fruit.

**Supplemental Movie S2.** X-ray computed microtomography scan of a mature endocarp horn.

**Supplemental Figure S1.** Raman intensity of compounds in the young endocarp tissue.

**Supplemental Figure S2.** Mixture analysis of cell wall spectrum C2.

**Supplemental Figure S3.** Raman imaging of washed endocarp tissue from green fruits.

**Supplemental Figure S4.** Raman spectrum of CaF_2_.

**Supplemental Figure S5.** Comparison of FT-IR spectra of mature nutshell tissues.

**Supplemental Figure S6.** Raman spectra of the extract and 2,3-Dimethylbenzoic acid.

**Supplemental Table S1.** Product information of reference compounds.
