# Supplementary material for: Hydrolyzable tannins are incorporated into the endocarp during sclerification of the water caltrop Trapa natans
Source: Plant Physiol. 2023 Jul 10;194(1):94–105. doi: 10.1093/plphys/kiad408 (PMC10762508; doi:10.1093/plphys/kiad408)
Supplement: kiad408_Supplementary_Data [file kiad408_supplementary_data.zip › SI_Hussetal_proof.pdf]

Supplementary Information for Huss et al.

Hydrolyzable tannins are incorporated into the endocarp during sclerification of the water caltrop *Trapa natans*

Supplemental Figures

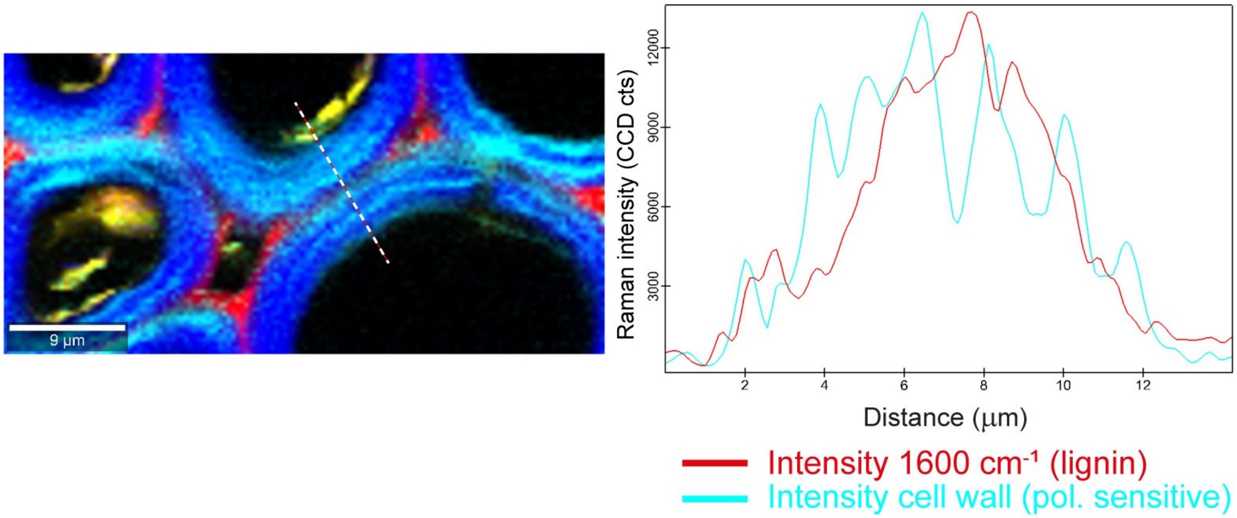

Fig. S1. Raman intensity of compounds in the young endocarp tissue: image shows the five identified components of Fig. 2 (zoom in the outer area) and the location of the line profile (white) across two neighboring cell walls. The plot shows the intensity of the lignin marker band at 1600 cm^{-1} (corresponding to C1) and the intensity of C3 (polarization sensitive cellulose band near 1096 cm^{-1}) as a function of distance along the line. The middle lamella is located at the maximum intensity of the 1600 cm^{-1} band. The transition between lamellae is characterized by strong intensity changes of the polarization sensitive cell wall component (i.e. cellulose) and smaller changes in lignification. Scale bar: 9 µm.

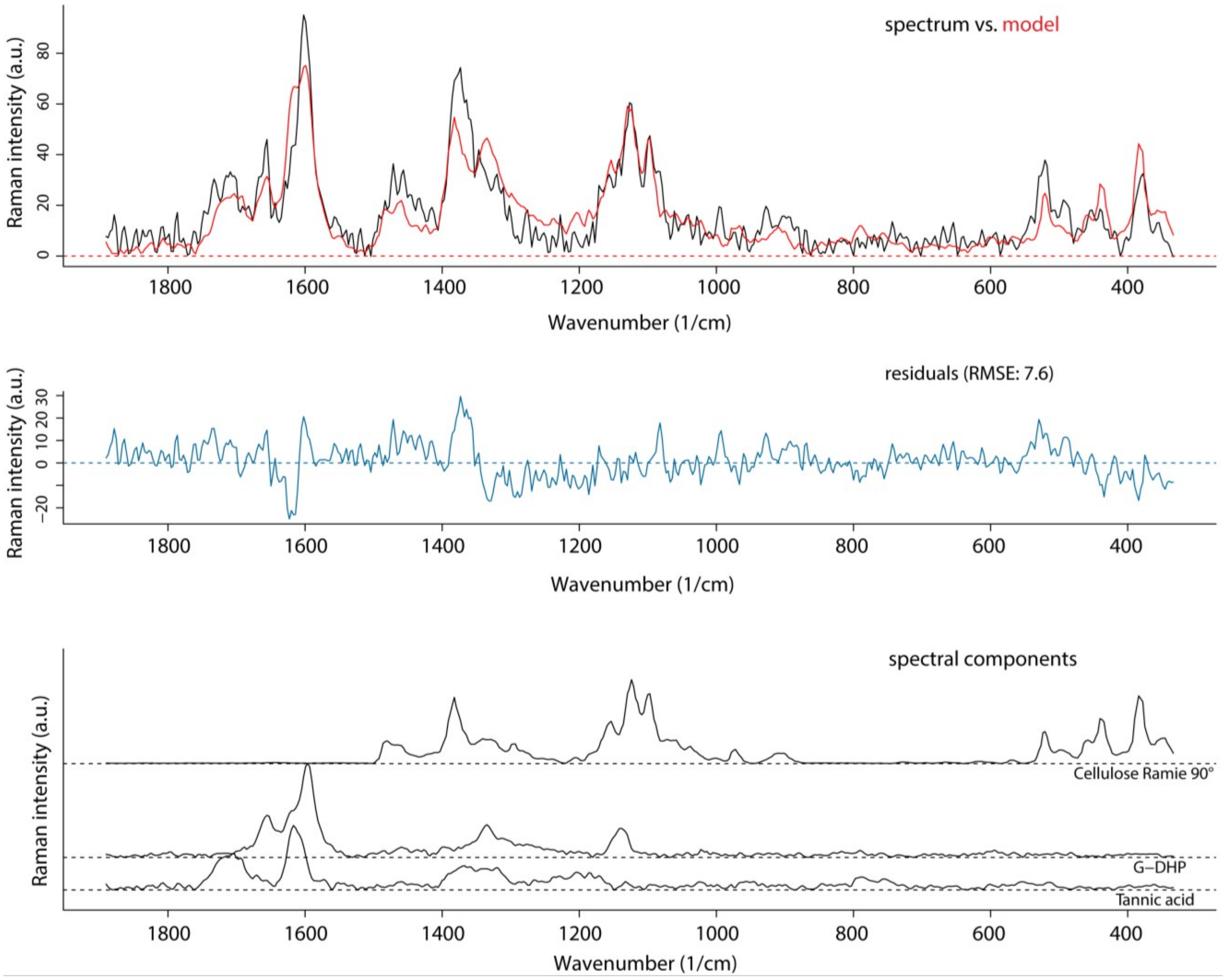

Fig. S2. Mixture analysis of cell wall spectrum C2 (CW I) shown in Fig. 2b. The model spectrum consists of a mixture of cellulose, lignin (G-DHP) and tannic acid and matches the original cell wall spectrum well. The presence of all three components has also been verified by histochemical staining (Fig. 2-3) and AFM-IR analysis (Fig. 4).

The algorithm of our in-house mixture analysis tool models the original spectrum by means of a linear combination of reference spectra using the Orthogonal Matching Pursuit (Pati *et al.*, 1993). A more detailed description of the mixture analysis tool can be found in Bock *et al*. (2021).

Pati, Y. C., Rezaiifar, R., and Krishnaprasad, P. S. (1993) Orthogonal matching pursuit: Recursive function approximation with applications to wavelet decomposition, in *Proceedings of 27th Asilomar conference on signals, systems and computers: IEEE*, (Pacific Grove: IEEE), 40–44.

Bock P., Felhofer M., Mayer K. and Gierlinger N. (2021) A Guide to Elucidate the Hidden Multicomponent Layered Structure of Plant Cuticles by Raman Imaging. *Front. Plant Sci*. **12**:793330.

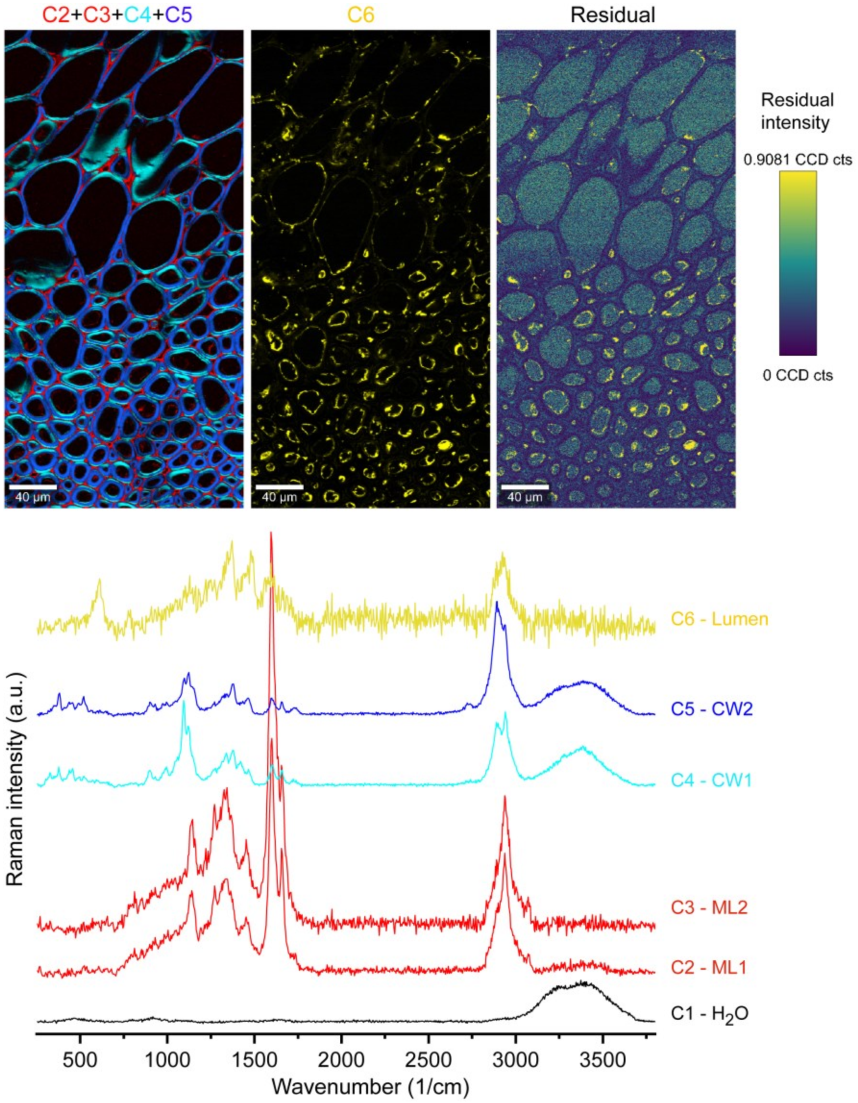

Fig. S3. Raman measurement of a washed endocarp section from green fruits (λ= 532 nm). Images and spectra were obtained via True Component Analysis. A total of six components were identified, which gave a low residual signal in cell walls. The residual is high in the lumen due to high fluorescence. Spectral features of the pink extract could not be identified with the same parameter settings during measurement and analysis, indicating the gallotannins have been removed successfully during washing with ethanol and water. Due to a very strong signal from cell corners compared to other parts of the middle lamella, the algorithm found two separate spectra that correspond to the middle lamella (C2 – ML1 and C3 – ML2). Spectra are shown with their original intensity. C: component, ML: middle lamella, CW: cell wall.

The True Component Analysis tool in WITec Project 5 Plus uses an algorithm that classifies the dataset based on a linear combination of the most different spectra (Dieing & Ibach, 2011). The analysis was started with 10 initial components and the number of components stepwise increased and decreased until the residual image was mostly homogeneous and reasonable images and spectra were obtained.

Dieing, T., and Ibach, W. (2011) Software Requirements and Data Analysis in Confocal Raman Microscopy, in *Confocal Raman Microscopy*, eds T. Dieing, O. Hollricher, and J. Toporski (Berlin: Springer), 61–89.

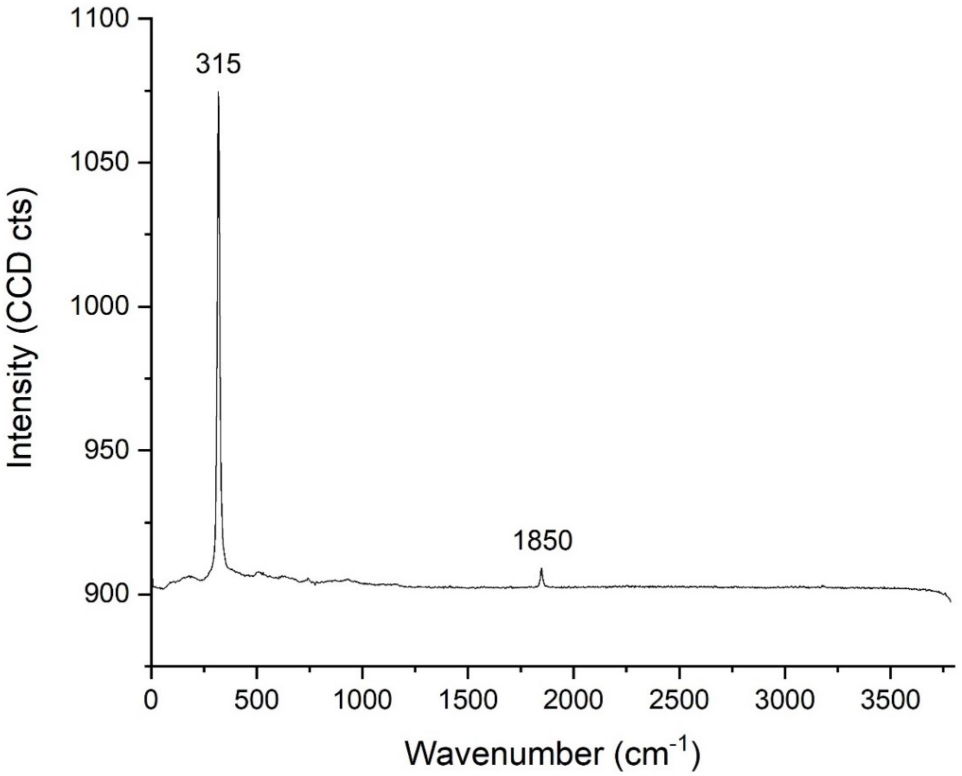

Fig. S4. Raman spectrum (λ=532nm) of the CaF_2 window that was used as a sample holder for Raman and FT-IR spectroscopy, showing a very strong, sharp band at 315 cm^{-1} and a smaller band at 1850 cm^{-1}. The spectrum was obtained from an image scan via cluster analysis. CCD: charge-coupled device.

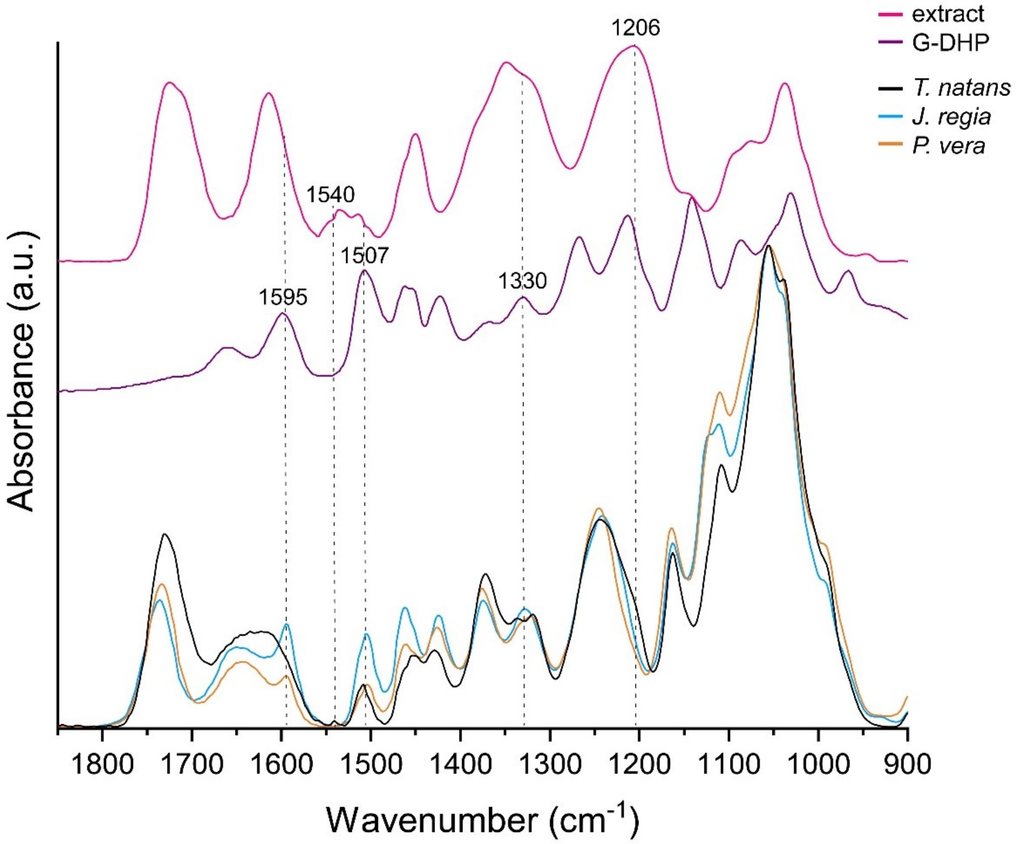

Fig. S5. Comparison of FT-IR spectra of mature nutshell tissues (*Trapa natans*, *Juglans regia*, *Pistacia vera*, min-max normalized) and two reference compounds (pink extract of *T. natans* and guaiacyl type dehydrogenative polymerization (G-DHP) lignin). The spectra of walnut (*J. regia*, average of 4 spectra) and pistachio (*P. vera*, average of 4 spectra) tissues show clear lignin bands at 1595 cm^{-1}, 1507 cm^{-1} and 1330 cm^{-1}, which are much weaker and/or overlap with bands of the extract in the spectrum of the endocarp tissue of *T. natans* (average of 8 spectra in L1-L2 of the horn region). Bands of the extract can be found at 1540 cm^{-1} (weak) and at 1206 cm^{-1} (shoulder) in the spectrum of *T. natans*, indicating that gallotannins (and/or galloyl glucose derivatives) are part of the tissue, i.e., cell walls. Due to the largely overlapping bands of the extract and G-DHP, it is not possible to draw clear conclusions on the lignin content of the endocarp of *T. natans*. For *J. regia* and *P. vera*, the band intensity at 1507 cm^{-1} can be used for a correlation with the lignin content when normalized to bands of cell wall polysaccharides, e.g. the cellulose band at 1056 cm^{-1} (lignin content *J. regia* > *P. vera*, also shown by Landucci *et al.*, 2020).

Landucci, L., Smith, R.A., Liu, S., Karlen, S.D. and Ralph, J. (2020) Eudicot nutshells: Cell-wall composition and biofuel feedstock potential. *Energy and Fuels*, **34**, 16274-16283.

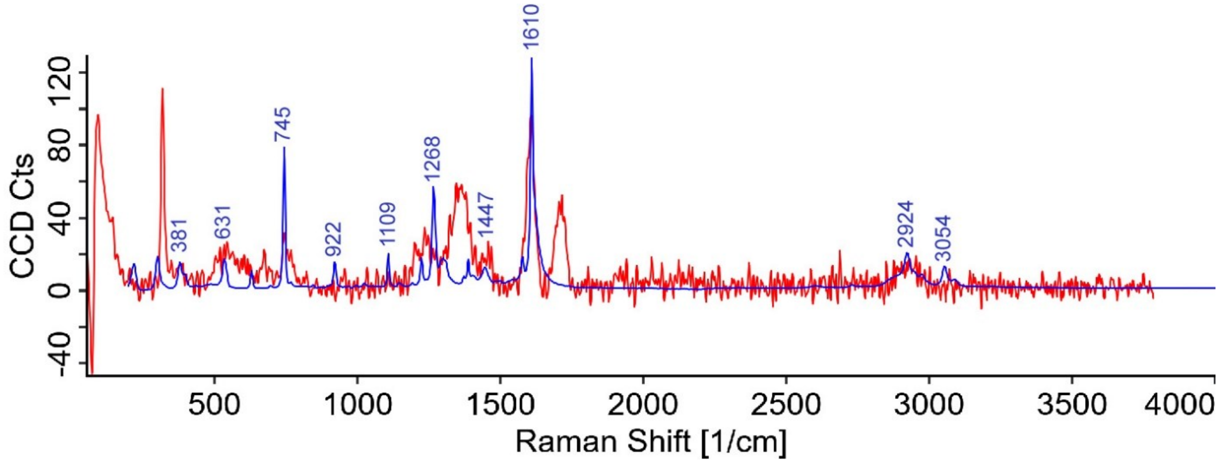

Fig. S6. Comparison of the Raman bands of the extract (red) and 2,3-Dimethylbenzoic acid (blue). The reference spectrum originates from the ST Japan 5.2 reference database of biochemicals (λ= 532 nm). CCD: charge-coupled device.

Supplemental Tables

Table S1. Product information of the reference compounds measured for FT-IR and Raman analysis.

Name	Producer	Product number
Tannic acid	Sigma Aldrich	403040
Gallic acid	Sigma Aldrich	8426490025
GA methylester	Sigma Aldrich	48640
GA ethylester	Sigma Aldrich	274197
Shikimic acid	Sigma Aldrich	S5375
Salicylic acid	Sigma Aldrich	247588
4-Hydroxybenzoic acid	Sigma Aldrich	240141
Benzoic acid	Sigma Aldrich	242381
G-DHP	UFT Tulln	G-DHP HA-Lig16
