# Supplementary material for: Hydrolyzable tannins are incorporated into the endocarp during sclerification of the water caltrop Trapa natans
Source: Plant Physiol. 2023 Jul 10;194(1):94–105. doi: 10.1093/plphys/kiad408 (PMC10762508; doi:10.1093/plphys/kiad408)
Supplement: kiad408_Supplementary_Data [file kiad408_supplementary_data.zip › Supplemental Movie Legends.pdf]

Supplemental Movie Legends for Huss et al.

Hydrolyzable tannins are incorporated into the endocarp during sclerification of the water caltrop *Trapa natans*

Movies

Movie S1. Cryo-stack of a green fruit showing the intense pink to purple coloration of the endocarp tissue. An entire fruit was embedded in ice and sliced from the pore towards the pedicel with a slice thickness of 100 μm.

Movie S2. Y-stack of an x-ray computed microtomography scan of a mature endocarp horn, showing the density gradient and vasculature (red arrows) within the tissue. Initially, all three tissue layers (L1-L3) and the seed are visible. Towards the spine tip, the tissue consists only of L1 and L2. Movie shows 50 frames per second.
